# Effect of Astaxanthin on the Expression and Activity of Aquaporin-3 in Skin in an In-Vitro Study

**DOI:** 10.3390/life10090193

**Published:** 2020-09-11

**Authors:** Nobutomo Ikarashi, Risako Kon, Chika Nagoya, Airi Ishikura, Yuri Sugiyama, Jiro Takahashi, Kiyoshi Sugiyama

**Affiliations:** 1 Department of Biomolecular Pharmacology, Hoshi University, 2-4-41 Ebara, Shinagawa-ku, Tokyo 142-8501, Japan; r-kon@hoshi.ac.jp; 2 Department of Clinical Pharmacokinetics, Hoshi University, 2-4-41 Ebara, Shinagawa-ku, Tokyo 142-8501, Japan; c.nagoya1226@gmail.com (C.N.); w.c.s.a1.cam@gmail.com (A.I.); snf-zeej63@ezweb.ne.jp (Y.S.); 3 Fuji Chemical Industries Co., Ltd., 1 Gohkakizawa, Kamiichi-machi, Nakaniikawa-gun, Toyama 930-0405, Japan; takahashi@fujichemical.co.jp; 4 Department of Functional Molecular Kinetics, Hoshi University, 2-4-41 Ebara, Shinagawa-ku, Tokyo 142-8501, Japan

**Keywords:** astaxanthin, aquaporin-3, keratinocytes, PHK16-0b cells, HaCaT cells, EpiSkin, glycerol permeability

## Abstract

Astaxanthin (3,3′-dihydroxy-β,β-carotene-4,4′-dione) is a red lipophilic pigment with strong antioxidant action. Oral or topical administration of astaxanthin has been reported to improve skin function, including increasing skin moisture. In this study, we examined the mechanism by which astaxanthin improves skin function by focusing on the water channel aquaporin-3 (AQP3), which plays important roles in maintaining skin moisture and function. When astaxanthin was added to PHK16-0b or HaCaT cells, the mRNA expression level of AQP3 increased significantly in a concentration-dependent manner in both cell lines. The AQP3 protein expression level was also confirmed to increase when astaxanthin was added to HaCaT cells. Similarly, when astaxanthin was added to 3D human epidermis model EpiSkin, AQP3 expression increased. Furthermore, when glycerol and astaxanthin were simultaneously added to EpiSkin, glycerol permeability increased significantly compared with that observed for the addition of glycerol alone. We demonstrated that astaxanthin increases AQP3 expression in the skin and enhances AQP3 activity. This result suggests that the increased AQP3 expression in the skin is associated with the increase in skin moisture by astaxanthin. Thus, we consider astaxanthin useful for treating dry skin caused by decreased AQP3 due to factors such as diabetes mellitus and aging.

## 1. Introduction

Astaxanthin (3,3′-dihydroxy-β,β-carotene-4,4′-dione) is a red lipophilic pigment contained in shrimp, salmon, etc. that has strong antioxidant action [1,2]. Astaxanthin ameliorates metabolic diseases, including insulin resistance [3,4] and obesity [5,6], and its usefulness as a functional food has been suggested. In addition, there have been reported various effects of astaxanthin on the skin [7,8,9,10,11,12,13]: increasing skin moisture and elasticity, inhibition of wrinkle development, and improving the epidermal barrier. Although these effects on the skin are likely associated with the antioxidant action of astaxanthin [14], many unanswered questions remain regarding the underlying mechanism.

Collagens, hyaluronic acid, and ceramides are moisturizing components in the skin, and many cosmetics targeting these substances are commercially available. Along with these components, the importance of aquaporins (AQPs) in mediating skin moisture is also attracting attention. Thirteen types of AQPs, numbered from AQP0 through AQP12, are expressed in various tissues in humans [15], and AQPs are known as passive transporters of water that are vital for water homeostasis [16]. Moreover, a subgroup of AQP water channels also facilitates transmembrane diffusion of small, polar solutes not only water: AQP3, AQP7, and AQP9 transport glycerol; AQP7, AQP9, and AQP10 transport urea; AQP1 transports carbon dioxide and so on [17,18]. As described above, AQP is a key molecule that is important for maintaining homeostasis in the living body, and its abnormality is known to cause various diseases. Of these AQPs, in the skin, AQP3 is highly expressed in keratinocyte, and a marked decrease in dermal water content and skin elasticity has been reported in AQP3 knockout mice [19,20,21]. Recently, research has clarified that the AQP3 level in the skin decreases when the skin dries due to psoriasis [22], vitiligo [23,24], diabetes mellitus [25], aging [26], etc. We previously confirmed that the dry skin that occurs with the administration of epidermal growth factor receptor (EGFR) tyrosine kinase inhibitors as anticancer agents is associated with decreased AQP3 levels in the skin [27]. Therefore, AQP3 is considered to play important roles in maintaining skin moisture and skin function. In this study, we attempted to clarify the mechanism by which astaxanthin improves skin function by focusing on AQP3 in the skin.

## 2. Materials and Methods

### 2.1. Materials

Astaxanthin was provided by Fuji Chemical Industries Co., Ltd. (Toyama, Japan). TRI reagent was purchased from Sigma-Aldrich Corp. (St. Louis, MO, USA). A high-capacity cDNA synthesis kit was purchased from Applied Biosystems (Foster City, CA, USA). RIPA (radioimmunoprecipitation assay) buffer and a protease inhibitor cocktail were purchased from Nacalai Tesque, Inc. (Kyoto, Japan). A rabbit anti-rat AQP3 antibody was purchased from Alomone Labs (Jerusalem, Israel). A mouse anti-rabbit glyceraldehyde-3-phosphate dehydrogenase (GAPDH) antibody was purchased from Merck Millipore (Darmstadt, Germany). A donkey anti-rabbit IgG-HRP (horseradish peroxidase) antibody, sheep anti-mouse IgG-HRP antibody, and enhanced chemiluminescence (ECL) Prime Western blotting detection reagents were purchased from GE Healthcare (Chicago, IL, USA). A glycerol colorimetric assay kit was purchased from Cayman Chemical (Ann Arbor, MI, USA).

### 2.2. PHK16-0b Cell Culture

Human keratinocytes PHK16-0b cells (Health Science Research Resources Bank, Osaka, Japan) were cultured in MCDB153 medium (5 µg/mL insulin, 0.5 µg/mL hydrocortisone, 10 µg/mL transferrin, 0.1 mM phosphorylethanolamine, 0.1 mM ethanolamine, 10 ng/mL epithelial growth factor, and 40 µg/mL bovine pituitary extract). PHK16-0b cells were plated and maintained in a subconfluent state. After dimethyl sulfoxide (DMSO; final concentration, 0.3%) or astaxanthin (2.5–10 μM) was added, the cells were incubated for 6 h.

### 2.3. HaCaT Cell Culture

Human keratinocytes HaCaT cells (Cell Lines Service, Eppelheim, Germany) were cultured in DMEM (dulbecco’s modified eagle medium) medium (100 μg/mL streptomycin, 100 U/mL penicillin G potassium, and 10% fetal bovine serum). HaCaT cells were plated and maintained in a subconfluent state. After DMSO or astaxanthin (2.5–10 μM) was added, the cells were incubated for 6 h or 24 h.

### 2.4. EpiSkin 3D Human Epidermis Model

The 3D human epidermis model EpiSkin was provided by EpiSkin SNC (Lyon, France). EpiSkin was cultured in medium provided by the manufacturer and treated on the apical side with DMSO or astaxanthin (10 μM) and incubated for 6 h, 24 h, 48 h, or 72 h.

### 2.5. Real-Time RT-PCR

RNA was prepared according to the procedure of Chomczynski and Sacchi [28] and chloroform and isopropanol was used for the extraction. A high-capacity cDNA synthesis kit was used to synthesize cDNA from 1 μg of RNA. Target gene expression was analyzed by real-time RT-PCR using the primers listed in Table 1. Target gene mRNA expression levels were estimated using the Delta-Delta Ct method and normalized to those of GAPDH. The expression level of GAPDH was stable under the conditions used in this study.

### 2.6. Preparation of Fractions from HaCaT Cells for Immunoblotting

HaCaT cells were lysed in RIPA buffer supplemented with a protease inhibitor cocktail. The lysate was sonicated and centrifuged (15,000× *g* for 15 min at 4 °C). The supernatant was analyzed by Western blotting.

### 2.7. Electrophoresis and Western Blotting

An equal amount of loading buffer (100 mM Tris, 20% glycerol, 0.004% bromophenol blue, 4% sodium dodecyl sulfate, and 10% 2-mercaptoethanol; pH 6.8) was added to the sample solution, and electrophoresed on a 12.5% (*w*/*v*) polyacrylamide gel (5 μg protein/lane). The protein was transferred to a polyvinylidene difluoride membrane and blocked with a skim milk solution for 1 h. The membrane was probed with primary antibody (1/500; 1 h) and secondary antibody (1/3000; 1 h). Recognized proteins were detected using ECL Prime detection reagent, and protein immunocomplexes were visualized using a Lumino Image Analyzer.

### 2.8. Glycerol Permeability Assay

EpiSkin was treated on the apical side with glycerol (final concentration, 100 µM) or a combination of glycerol and astaxanthin (final concentration, 10 µM). Samples were collected from the basal side up to 72 h after treatment, and the concentration of glycerol was determined using a glycerol colorimetric assay kit.

### 2.9. Statistical Analysis

Numerical data are expressed as the means ± standard deviations (SDs). Comparisons between two groups were made using a Student’s t-test. For comparisons between multiple groups, ANOVA with Bonferroni correction was used.

## 3. Results

### 3.1. Effect of Astaxanthin on the mRNA Expression Levels of AQP3 in PHK16-0b and HaCaT Cells

Astaxanthin was added to PHK16-0b and HaCaT human keratinocytes, and the mRNA expression levels of AQP3 were measured after 6 h.

**Figure 1:**
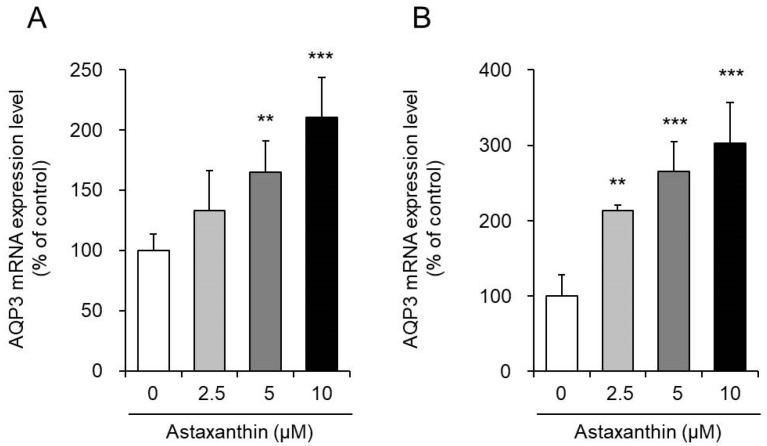
Effect of astaxanthin on the mRNA expression level of aquaporin-3 (AQP3) in PHK16-0b (**A**) and HaCaT (**B**) cells. Astaxanthin was added to PHK16-0b or HaCaT cells, and the cells were incubated for 6 h. The AQP3 mRNA expression level was measured by real-time RT-PCR. Expression levels were normalized to those of glyceraldehyde-3-phosphate dehydrogenase (GAPDH), and the data are presented as the mean values as a percentage of the control values, which were set to 100% (mean ± SD, *n* = 4; ** *p* < 0.01 and *** *p* < 0.001).

When astaxanthin was added to PHK16-0b cells, the mRNA expression level of AQP3 increased significantly in a concentration-dependent manner (Figure 1A). Similarly, in HaCaT cells, the mRNA expression level of AQP3 increased in a concentration-dependent manner after the addition of astaxanthin. In particular, when astaxanthin was added at a concentration of 10 μM, the mRNA expression of AQP3 increased significantly to a level nearly threefold higher than that in the corresponding control cells (Figure 1B). However, 24 h after the addition of astaxanthin to HaCaT cells, the AQP3 mRNA expression level returned to the control level (data not shown).

The above findings demonstrate that astaxanthin enhances the transcription of AQP3.

### 3.2. Effect of Astaxanthin on the Protein Expression Level of AQP3 in HaCaT Cells

Astaxanthin was added to HaCaT cells, and the protein expression level of AQP3 was measured after 24 h.

**Figure 2:**
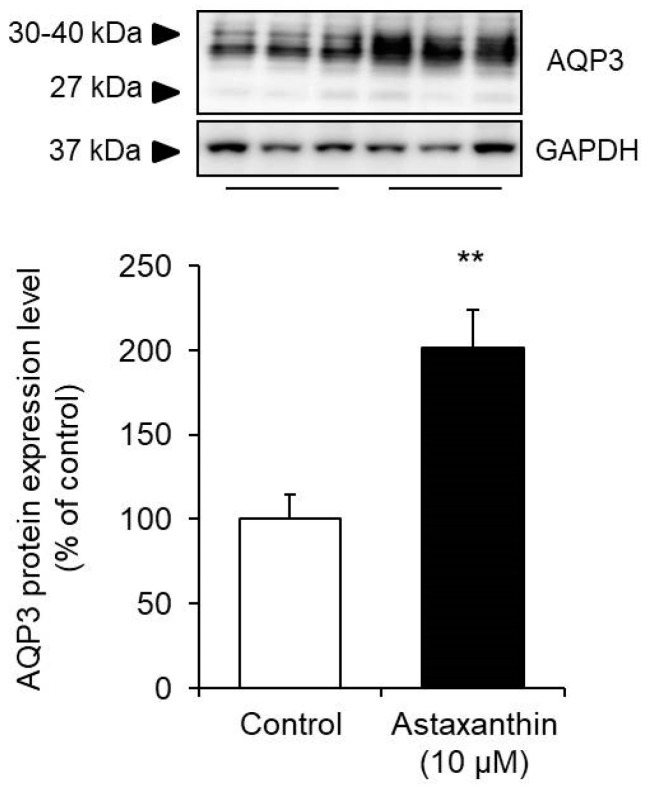
Effect of astaxanthin on the protein expression level of AQP3 in HaCaT cells. Astaxanthin was added to HaCaT cells, and the cells were incubated for 24 h. The AQP3 protein expression in HaCaT cells was analyzed by Western blotting. Expression levels were normalized to those of GAPDH, the data are shown as the mean values as a percentage of the control values, which were set to 100% (mean ± SD, *n* = 5; ** *p* < 0.01).

The protein expression level was measured by Western blotting, and the signal bands of AQP3 were detected at approximately 27 kDa and 30 to 40 kDa, which have been reported to correspond to nonglycosylated (27 kDa) and glycosylated (30 to 40 kDa) AQP3 [29,30]. The presence or absence of glycosylation results in differences in AQP stability and migration to the cell membrane but does not affect their water permeability functions [31,32,33]. Therefore, in this study, the sum of these bands was analyzed as the expression level of AQP3. When astaxanthin was added to HaCaT cells, the protein expression of AQP3 increased significantly to a level nearly twofold higher than that in the corresponding control cells (Figure 2).

The above findings demonstrate that astaxanthin increases the protein expression level, in addition to the mRNA expression level, of AQP3.

### 3.3. Effect of Astaxanthin on Glycerol Permeability via AQP3 in the EpiSkin Epidermal System

AQP3 transports glycerol in addition to water [34]. We assessed the effect of astaxanthin on glycerol permeability via AQP3 in the EpiSkin epidermal system. The EpiSkin epidermal system is a reconstituted organotypic culture of human keratinocytes forming a multilayer differentiated epidermis on a collagen matrix [35,36].

**Figure 3:**
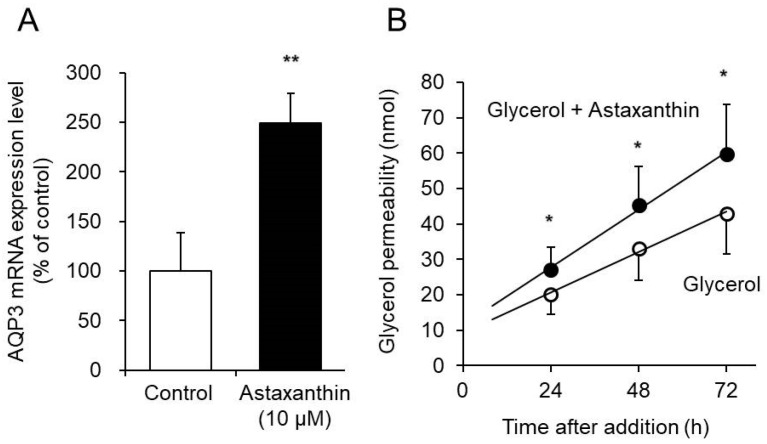
Effect of astaxanthin on glycerol permeability via AQP3. (**A**) Astaxanthin was added to EpiSkin and incubated for 6 h. The AQP3 mRNA expression level was measured by real-time RT-PCR. The expression level was normalized to that of GAPDH, and the data are presented as the mean value as a percentage of the control value, which was set to 100%. (**B**) EpiSkin was treated on the apical side with glycerol or a combination of glycerol and astaxanthin. Samples were collected from the basal side 24 h, 48 h, and 72 h after treatment, and the concentration of glycerol was determined (mean ± SD, *n* = 6; * *p* < 0.05 and ** *p* < 0.01).

When astaxanthin was added to EpiSkin, the mRNA expression of AQP3 increased significantly, to a level nearly 2.5-fold higher than that in the control group (Figure 3A). Similar results were obtained when astaxanthin was added to PHK16-0b and HaCaT cells. Simultaneous addition of glycerol and astaxanthin to EpiSkin significantly increased glycerol permeability compared with that resulting from the addition of glycerol alone (Figure 3B).

These results demonstrate that astaxanthin increases the expression of AQP3 in EpiSkin and enhances glycerol permeability.

## 4. Conclusions

Water channel AQPs have been validated as an important drug target but there is no single drug that has yet been approved to successfully target them [37,38]. Therefore, the search for a drug targeting AQP is very important, and much research has been done in the world. Oral or topical administration of astaxanthin has been reported to improve skin function, including an increase in skin moisture [7,8,9,10,11,12,13]. In this study, we examined the mechanism by which astaxanthin improves skin function by focusing on AQP3, which plays important roles in maintaining skin moisture and function.

PHK16-0b is an immortalized foreskin epidermal keratinocyte cell line transformed with human papilloma virus (HPV) 16 and which expresses AQP3 [39,40]. HaCaT cells are a transformed immortal keratinocyte cell line used widely in scientific research on AQP3 [41,42]. When astaxanthin was added to PHK16-0b and HaCaT cells, the mRNA expression level of AQP3 increased significantly in a concentration-dependent manner in both cell lines (Figure 1). In addition, an increase in the protein expression level of AQP3 was also confirmed (Figure 2). Furthermore, astaxanthin increased glycerol permeability in the EpiSkin epidermal system (Figure 3). These results suggest that astaxanthin enhances AQP3 activity through an increase in AQP3 expression. As previous studies have reported a decrease in skin moisture in AQP3 knockout mice [19,20,21], substances that increase AQP3 in the skin are considered to be useful as skin moisturizers. The present study suggests that the increased expression and activity of AQP3 is involved in the skin moisturizing action of astaxanthin, in addition to its antioxidant effect [14].

Astaxanthin acts as a peroxisome proliferator-activated receptor-gamma (PPARγ) modulator and induces the expression of liver X receptor (LXR) via PPARγ activation [43,44]. In addition, recent studies have demonstrated that ligands of either PPARγ or LXR increase the expression level of AQP3 in epidermal keratinocytes [45,46,47]. These results suggest that astaxanthin enhances the transcription of AQP3 via its PPARγ agonist action and increases AQP3 protein expression, thereby enhancing AQP3 activity.

The rate of carotenoid absorption from the digestive tract is low. However, oral administration of astaxanthin to mice was reported to increase both the blood concentration and skin content of astaxanthin [13]. Thus, we believe that the demonstrated effects of the increased AQP3 expression level in the skin will occur with either oral or topical administration.

As mentioned above, AQP has subfamilies and is widely distributed throughout the body. It was examined whether the AQP3 increasing effect by astaxanthin was similarly observed on cells of other tissues. As a result, it was found that astaxanthin significantly increased the mRNA expression level of AQP3 in human colon cancer HT-29 cell line (data not shown). AQP3 in the large intestine is important for regulation of fecal water content [48]. Therefore, it was considered that astaxanthin may be a useful substance for abnormal water metabolism in the digestive tract.

It was reported that the AQP3 level in the skin decreases when the skin dries due to psoriasis [22], vitiligo [23,24], diabetes mellitus [25], aging [26], and anti-cancer drugs [27]. This result suggests that the increased AQP3 expression in the skin is associated with the increase in skin moisture by astaxanthin. Thus, we consider astaxanthin useful for treating dry skin caused by decreased AQP3 due to factors such as diabetes mellitus and aging etc. Further developments in this research area are expected.

## Figures and Tables

**Table 1:** Primer sequences used for real-time PCR.

Target	Forward Primer (5′ to 3′)	Reverse Primer (5′ to 3′)
AQP3	AGACAGCCCCTTCAGGATTT	TCCCTTGCCCTGAATATCTG
GAPDH	ATGGGGAAGGTGAAGGTCG	GGGGTCATTGATGGCAACAATA
